# Bath additives for the treatment of childhood eczema (BATHE): protocol for multicentre parallel group randomised trial

**DOI:** 10.1136/bmjopen-2015-009575

**Published:** 2015-10-30

**Authors:** Miriam Santer, Kate Rumsby, Matthew J Ridd, Nick A Francis, Beth Stuart, Maria Chorozoglou, Wendy Wood, Amanda Roberts, Kim S Thomas, Hywel C Williams, Paul Little

**Affiliations:** 1 Department of Primary Care and Population Sciences, University of Southampton, Southampton, UK; 2 Department of School of Social & Community Medicine, University of Bristol, Bristol, UK; 3 Wales School of Primary Care Research, Cardiff University, Cardiff, UK; 4 Southampton Health Technology Assessment Centre (SHTAC), University of Southampton, Southampton, UK; 5 Southampton Clinical Trials Unit, University of Southampton, Southampton, UK; 6 Centre of Evidence-Based Dermatology, University of Nottingham, Nottingham, UK

**Keywords:** PRIMARY CARE, DERMATOLOGY

## Abstract

**Introduction:**

Bath emollients are widely prescribed for childhood eczema, yet evidence of their benefits over direct application of emollients is lacking. *Objectives* To determine the clinical and cost-effectiveness of adding bath emollient to the standard management of eczema in children

**Methods and analysis:**

*Design*: Pragmatic open 2-armed parallel group randomised controlled trial. *Setting*: General practitioner (GP) practices in England and Wales. *Participants*: Children aged over 12 months and less than 12 years with eczema, excluding inactive or very mild eczema (5 or less on Nottingham Eczema Severity Scale). *Interventions*: Children will be randomised to either bath emollients plus standard eczema care or standard eczema care only. *Outcome measures*: Primary outcome is long-term eczema severity, measured by the Patient-Oriented Eczema Measure (POEM) repeated weekly for 16 weeks. Secondary outcomes include: number of eczema exacerbations resulting in healthcare consultations over 1 year; eczema severity over 1 year; disease-specific and generic quality of life; medication use and healthcare resource use; cost-effectiveness. Aiming to detect a mean difference between groups of 2.0 (SD 7.0) in weekly POEM scores over 16 weeks (significance 0.05, power 0.9), allowing for 20% loss to follow-up, gives a total sample size of 423 children. We will use repeated measures analysis of covariance, or a mixed model, to analyse weekly POEM scores. We will control for possible confounders, including baseline eczema severity and child's age. Cost-effectiveness analysis will be carried out from a National Health Service (NHS) perspective.

**Ethics and dissemination:**

This protocol was approved by Newcastle and North Tyneside 1 NRES committee 14/NE/0098. Follow-up will be completed in 2017. Findings will be disseminated to participants and carers, the public, dermatology and primary care journals, guideline developers and decision-makers.

**Trial registration number:**

ISRCTN84102309.

**Strengths and limitations of this study**

- We are carrying out the first large trial to provide evidence about the clinical and cost-effectiveness of bath emollients in the treatment of childhood eczema.
- Children will be randomised to either bath emollients plus standard eczema care or standard eczema care only.
- Primary outcome is long-term eczema severity, measured by the Patient-Oriented Eczema Measure repeated weekly for 16 weeks.
- The trial is ‘open label’ as it would not be possible to create a convincing placebo for bath emollients, which many carers and children are already familiar with.

## Background

Childhood eczema is very common, affecting over 20% of children aged 5 years or under at some point.^1^ Eczema can cause significant distress to children and their families due to sleep disturbance and itch.^2^ ^3^ Health and societal costs of eczema are thought to cause a similar economic burden to that for asthma.^4^ ^5^ The term atopic eczema (synonymous with atopic dermatitis) is widely used to denote a clinical phenotype, rather than those who are truly atopic defined by the presence of IgE-specific antibodies to common environmental allergens. In this study, we use the term ‘eczema’ throughout to refer to the ‘atopic eczema’ clinical phenotype, in accordance with the recommended nomenclature of the World Allergy Organisation.^6^

Guidelines suggest that emollients form the mainstay of treatment for eczema and should be used regularly by all patients with other treatments, such as topical corticosteroids, used in addition where necessary.^7^ Emollients are thought to act by providing a protective layer over the skin, decreasing moisture loss and occluding against irritants. There are three methods of application of emollients: (1) leave-on (directly applied) emollients, where emollients are applied to the skin and left to soak in; (2) soap substitutes, where emollients are used instead of soap or other washing products; and (3) bath emollients (or bath additives), which are oil and/or emulsifiers designed to disperse in the bath. All three approaches are often used together.

While there is widespread clinical consensus on the need for leave-on emollients and soap substitutes, there is less agreement regarding the additional benefits of bath emollients. Despite this, they are widely prescribed at a cost of nearly £25 million per year to the National Health Service (NHS) in England.^8^ A previous systematic review has revealed no convincing evidence for the use of bath emollients in the treatment of eczema.^9^ ^10^ Available data consist of isolated case series and case reports, with no controlled studies. No relevant trials have been published since 2007^11^ and trial registries reveal no ongoing studies.

In addition to concerns about cost-effectiveness, potential harms from using bath emollients include skin irritation and greasier bath surfaces that can increase the risk of slips and accidents (listed in the Summary of Product Characteristics of leading brands). There is also a concern that people who use bath emollients in place of leave-on emollients are receiving substandard emollient therapy.^10^

The effectiveness of adding antiseptic agents to bath emollients has also not been demonstrated. Two small randomised studies^12^ ^13^ compared ‘bath emollient’ with ‘bath emollient plus antiseptic’ on a range of outcomes, but there were no significant differences between groups, including colony counts of *Staphylococcus aureus*.^14^ For this reason, we chose to exclude bath emollients which incorporate an antiseptic, because of the absence of benefit and possible increased risk of skin irritation.^15^

Pragmatic clinical trials aim to test the effectiveness of an intervention in a real-life setting in order to recruit a study population that is as similar as possible to the population on which the intervention is meant to be used. Whereas an explanatory clinical trial aims to answer the question, ‘Can this intervention work under ideal conditions?’ a pragmatic approach seeks to answer the question, ‘Does this intervention work under usual conditions?’^16^ ^17^ Features of pragmatic trials include: that they use clinically important outcomes, commonly participant-reported outcomes; that they include longer term follow-up; and that participants are encouraged to adhere to the intervention only to the extent that would be anticipated in usual care.

Although relatively few pragmatic trials have been carried out in dermatology,^18^ we felt that a definitive pragmatic clinical trial, including outcomes of relevance to participants and including long-term follow-up, was the most appropriate design to address the question of the effectiveness of bath emollients in addition to standard eczema care in everyday care. We chose an ‘open label’ design as it would not be possible to create a convincing placebo for bath emollients, which make the bath feel ‘greasy’. We wished to design a trial with a clinical outcome relevant to participants. In eczema, the appearance of the skin does not always closely reflect symptoms causing a major impact on the child and family, such as sleep disturbance and itch.^19^ It was therefore particularly important to design a trial with a validated participant-reported primary outcome.

We aim to determine the clinical and cost-effectiveness of adding bath emollient to the standard management of atopic eczema in children, which includes regular application of leave-on emollients with use of topical corticosteroids as required. The BATHE trial is a pragmatic randomised open-label multicentre superiority trial with two parallel groups and a primary outcome of long-term control as measured by Patient-Oriented Eczema Measure (POEM) weekly scores over 16 weeks. Children will be randomised in a 1:1 ratio to either bath emollients plus standard eczema care or standard eczema care only. This paper outlines the study protocol, in accordance with the SPIRIT guidelines (Standard protocol items; recommendations for interventional trials).^20^

## Methods

### Study setting

The BATHE study will recruit participants registered at approximately 100 general practices in Wales, West of England and South-West England (list of practices will be on study website when available). Practices will be recruited through the local Clinical Research Networks in England and The National Institute for Social Care and Health Research in Wales. We aim to recruit practices that are broadly representative of UK primary care in terms of practice size and sociodemographics.

### Eligibility

Children are eligible to participate in the study if they are aged over 12 months and less than 12 years with eczema according to UK Diagnostic Criteria for Atopic Eczema.^21^ We will exclude children with inactive or very mild eczema, defined as a score of 5 or less on Nottingham Eczema Severity Scale (NESS),^22^ in order to avoid floor effects. We will exclude children who usually have a bath less than once per week.

**Figure 1:**
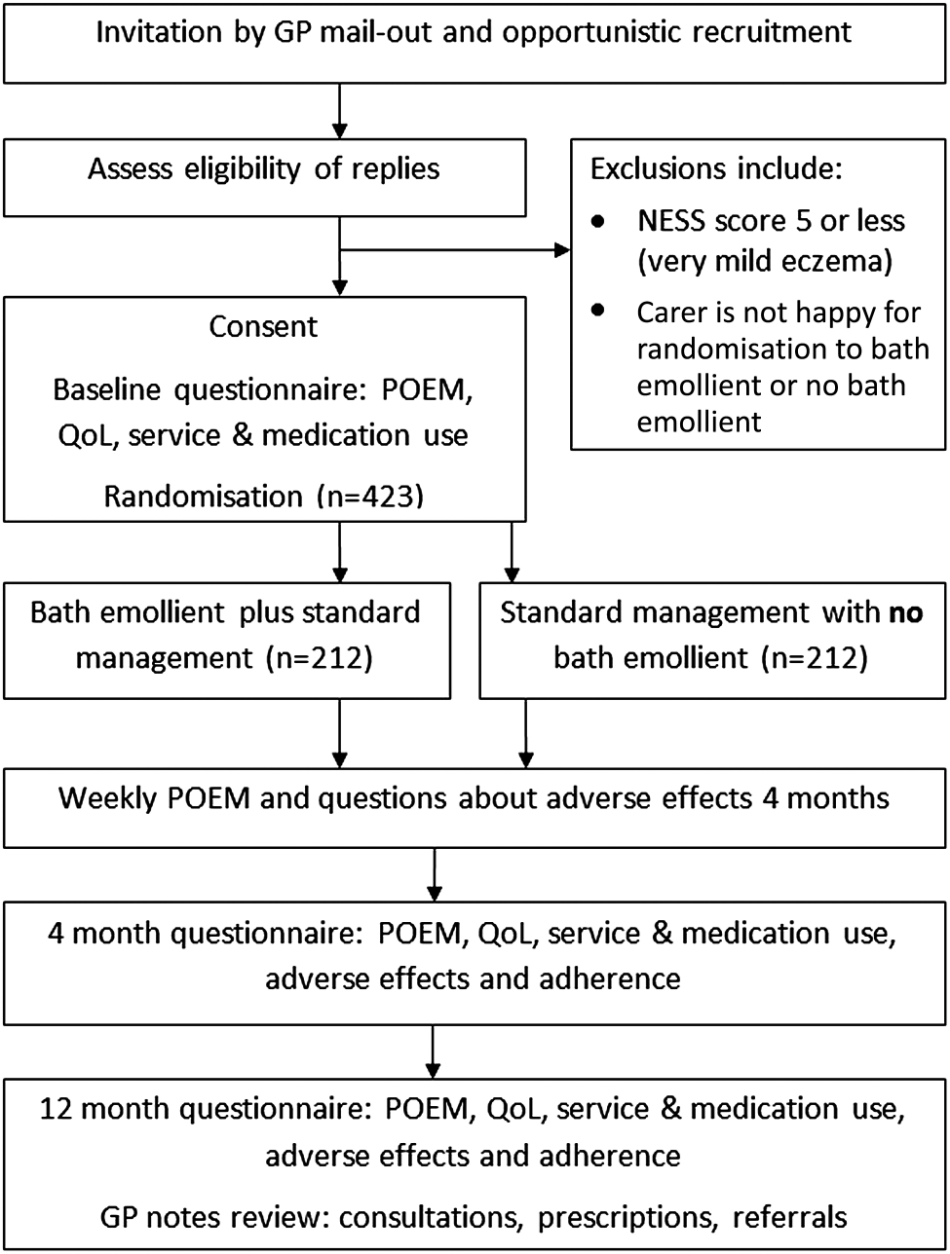
Flow of participants through the trial (GP, general practitioner; NESS, Nottingham Eczema Severity Scale; POEM, Patient-Oriented Eczema Measure; QoL, quality of life).

Children will be excluded if they or their carers are not prepared to be randomised to either intervention group or if their carer is unable to give informed consent or does not have sufficient English to complete the trial documentation. Children will be excluded if they are currently participating in any other clinical trial. If a family has more than one child who meets the eligibility criteria, they will be asked to choose just one child to participate in the trial, as it would be burdensome to have different bathing regimens within one family, as many will bath their children together. See figure 1 for participant timeline.

### Interventions

Eligible children will be randomised 1:1 to either bath emollients plus standard eczema care or standard eczema care only. Standard eczema care in this study constitutes usual general practitioner (GP) care, supplemented by evidence-based guidance. We will provide basic information on eczema care at baseline to participants and GPs in the form of a booklet based on the National Institute for Health and Care Excellence (NICE) guidance^7^ (available on study website). Standard care includes advice to regularly use leave-on emollients plus intermittent topical corticosteroids if required. Standard care of eczema in the UK is generally delivered through 10 min consultations in primary care initiated by carers. GPs in the study will continue to refer to dermatology services when they feel this to be necessary. In the UK, referral criteria include: diagnostic uncertainty; eczema not satisfactorily controlled; child or carer may benefit from specialist advice on treatment application.^7^

Baseline appointments will be carried out by a trial coordinator at the participant's GP surgery (or at their home if surgery premises not available) and informed consent/assent will be sought at this point. At the baseline appointment, both groups will be given basic information about eczema and will be given identical verbal and written information on how to wash children with eczema as soap avoidance is an important component of eczema care. This information is also available on the study website. We will ask all carers to either wash their child with water alone or to use a leave-on emollient as a soap substitute. If they have difficulties with this and request to use an emollient wash product then they may do so. Guidelines on frequency of bathing vary, but most healthcare professionals advise daily bathing for children with eczema^23^ and this advice is offered in our information to carers.

Children allocated to receive bath emollients will receive a prescription for a bath emollient from a GP in the participating practice and we will ask the prescriber to enter this as a ‘repeat’ prescription, so that carers may obtain further supplies as necessary, reflecting usual prescribing in UK primary care. Carers will be asked to use bath emollients as prescribed or described on the packaging, to reflect how they are used in usual practice.

The British National Formulary (BNF) lists 13 different bath emollients, but in clinical practice, a few of these are commonly prescribed. We will encourage participating practices to prescribe one of the following products: Oilatum Fragrance Free Junior, Balneum bath oil or Aveeno Bath Oil. These products account for the majority of bath emollient prescriptions issued in the UK^8^ and appear in local prescribing formularies for participating centres. Participants may express a preference for one brand of bath emollient over another, or if no preference is expressed, the choice of prescription will be determined by the GP. If children or carers wish to change bath emollient during the trial then they may change to an alternative within the three above, or choose a different bath emollient if their GP is happy to prescribe this. Prescribers will be asked to avoid bath emollients that contain additional ingredients such as antipruritics and antiseptics.

### Intervention adherence

We will ask children and their carers to adhere to their treatment allocation for a year. If they feel that the eczema is deteriorating, we will ask carers to consult their GP in the usual way in order to address whether other treatments are necessary. We will encourage adherence to treatment allocation by ensuring that participating general practices are committed to supporting the study and receive clear advice regarding maintaining treatment allocation wherever possible. As this is a pragmatic trial we wish to evaluate the benefit of emollient use in everyday practice and, for this reason, will not make additional efforts to ensure adherence to treatment allocation in either group. We will assess adherence to treatment allocation in questionnaires.

### Outcomes

#### Primary outcome measure

Our primary outcome measure is difference between the two treatment groups in long-term control as measured by POEM scores captured weekly over the first 16 weeks. POEM is a patient-reported outcome based on symptoms over the previous week which can be completed by the child's carer or the child themselves.^24^ POEM is the only patient-reported outcome that demonstrated sufficient validity and repeatability in a systematic review of outcome measures for eczema^25^ and has been recommended as the preferred instrument for capturing patient-reported symptoms in eczema trials by the Harmonizing Outcome Measures for Eczema initiative (http://www.homeforeczema.org). Our primary outcome measure is based on repeated measures of POEM data collected weekly over 16 weeks because this reflects the impact of this relapsing and remitting chronic condition better than comparing outcomes at a single follow-up point. However, if weekly questionnaires prove too burdensome for participants and there is excessive missing data, we will consider change in POEM score from baseline to 4 months as our primary outcome.

Because of the burden of weekly questionnaires on participants, we have limited weekly data collection to the first 16 weeks of the trial for the primary outcome assessment but follow-up will continue for 12 months. Participants may choose to complete questionnaires either online or by post. See table 1 for schedule of observations.

**Table 1:** Schedule of observations

Outcomes collected	Prescreen	Baseline	Weekly for 15 weeks	16 weeks	4 weekly for 32 weeks	52 weeks
Carer-reported outcomes						
UK diagnostic criteria for eczema	✓	✓				
Eczema severity over past year (NESS)	✓					
Demographics		✓				
Prior belief in bath emollients	✓	✓		✓		✓
Service use		✓		✓	✓	✓
Medication use		✓		✓	✓	✓
Eczema severity over past week (POEM)		✓	✓	✓	✓	✓
Eczema-related quality of life (DFI)		✓		✓		✓
Generic quality of life (CHU-9D)		✓		✓		✓
Questions about washing		✓		✓		✓
Adverse effects from bathing		✓	✓	✓	✓	✓
Adherence/avoidance of bath emollients	✓	✓		✓		✓
Review of GP records for 12 months from recruitment						
Number of consultations*						✓
Referrals for eczema						✓
Medication use						✓

*Consultations will be categorised into the following four groups:

▸ Consultation record mentions flare of eczema or infected eczema or prescription or advice to use topical corticosteroids/calcineurin inhibitors or antibiotics (topical or oral antibiotics for skin).

▸ Consultation record mentions eczema but with no indication that this was an eczema flare or infected eczema.

▸ Consultation record mentions skin rash, itch or dryness mentioned but no mention of eczema.

▸ Consultation recorded with no mention of eczema or skin rash, itch or dryness.

CHU-9D, Child Health Utility 9D; DFI, Dermatitis Family Impact; GP, general practitioner; NESS, Nottingham Eczema Severity Scale; POEM, Patient-Oriented Eczema Measure.

#### Secondary outcome measures

Secondary outcomes include differences between groups in POEM scores captured monthly over 12 months.

We will analyse differences between groups in number of eczema exacerbations resulting in a primary healthcare consultation over 12 months measured by GP notes review. Exacerbations will be defined as consultations where there is mention of eczema and topical steroid or topical calcineurin inhibitor has been advised or prescribed (see table 1 schedule of observations for further detail). We will also measure dermatology referrals and prescribing for eczema over 12 months by GP notes review. We will assess service use and medication use by carer report, in addition to GP notes review.

We will ask both groups about use of bath emollient in order to assess adherence to treatment allocation. Adverse effects of bathing, such as stinging in the bath or slipping in the bath or bathroom will also be asked in both groups to allow exploration of any differences between groups.

Differences between groups in change from baseline in disease-specific quality of life at 4 and 12 months will be measured by Dermatitis Family Impact (DFI). DFI^26^ is a widely used validated instrument measuring impact of eczema on the family's quality of life.

We will measure differences between groups in change from baseline in generic quality of life at 4 and 12 months, using the Child Health Utility 9D (CHU-9D).^27^ The use of EQ-5D in children has been questioned and it does not capture quality of life issues pertinent to childhood eczema. The CHU-9D is a paediatric generic preference-based utility measure exclusively developed with children aged 7–11 years and is more suitable for capturing quality of life impact related to eczema, such as sleep disturbance and child's mood. Personal communication with the team who developed this measure confirmed that studies are underway trialling its application in children aged 5–7 years but, to our knowledge, there are no studies reporting for infants. There are no suitable utility measures validated for very young children aged 1–4 years, but the CHU-9D performed well in a similar population in the SPaCE feasibility trial (CHU-9D data currently being prepared for publication).^28^

We will use healthcare resource use data in order to cost the intervention, collected by practice records, and data collected by carer/parent-completed questionnaires based on the modified Client Service Receipt Inventory (CSRI).^29^

### Sample size

The sample size was calculated for repeated measures analysis of covariance (ANCOVA) in weekly POEM scores over 16 weeks. Using data from a similar population in the SWET trial,^30^ we aim to detect a mean difference of 2.0 (SD 7.0) between intervention and control groups. An α of 0.05 and power 0.9 gives a sample size of 338. Allowing for 20% loss to follow-up, this gives a total sample size of 423 children.

### Recruitment

Postal invitations to participate will be sent to the parent/carer of children aged over 12 months and less than 12 years who, by means of a search of their electronic medical records, are identified as having a diagnosis of eczema and who have obtained one or more prescriptions for drugs acting on the skin over the previous 12 months (as a recent prescription would suggest that the eczema is still active). Invitation packs may also be given opportunistically by health professionals to carers of children meeting these criteria.

Invitation packs contain an invitation letter on GP-headed notepaper, participant information sheet, a brief screening questionnaire and a reply slip to return to the study team. The brief screening questionnaire includes the NESS^22^ and questions to check that they meet UK diagnostic criteria for eczema.^21^ The study team will then contact carers to discuss the study further and to confirm eligibility criteria, before inviting them to a recruitment appointment with a clinical studies officer at either their home or their GP practice.

Previous experience from the SPaCE feasibility trial^28^ suggests that we will obtain approximately seven participants from each general practice, and that approximately 60 practices will be sufficient to recruit to target. We will increase or decrease the number of practices participating on the basis of response rates to mail-outs over the first few months of recruitment.

## Methods

### Assignment of interventions

Participants will be randomly allocated to treatment group using computer-generated random sequence, stratifying by region. Bath emollients make the bath feel ‘greasy’, and it is therefore not possible to make a convincing placebo, particularly as a survey carried out prior to the study suggested that many families of children with eczema have experience of using bath emollient (see table 2). As discussed above, the main focus for this pragmatic study is patient-centred, and primary outcome is therefore a participant/proxy-reported score. Care providers and clinical studies officers will not be blinded as they are involved in ensuring participants receive the correct treatment and may be involved in discussing adherence with participants over the course of the year. Furthermore, the additional staff cost that would have been incurred by having researchers ‘blind’ to group allocation did not seem justifiable if the primary outcome is participant-reported. The trial statistician will remain blinded to treatment allocation.

**Table 2:** Pretrial survey findings

How many times a week does your child have a bath? (n=211)		
Less than once a week	19	(9%)
1–2 times per week	47	(22.3%)
3–4 times per week	57	(27%)
5–6 times per week	25	(11.8%)
7 times per week	55	(26.1%)
More than 7 times per week	4	(1.9%)
How many times a week does your child have a shower? (n=211)		
Less than once a week	127	(60.2%)
1–2 times per week	39	(18.5%)
3–4 times per week	19	(9%)
5–6 times per week	5	(2.4%)
7 times per week	7	(3.3%)
More than 7 times per week	2	(0.9%)
What do you use to wash your child's face, hands and body? (n=209)		
Emollient moisturiser	62	29.7%
Bath emollient	128	61.2%
Soap substitute	19	9.1%
Normal soap or body wash	21	10%
Water only	40	19.1%
Other	22	10.5%
What do you use to wash your child's hair? (n=209)		
Emollient moisturiser	7	3.3%
Bath emollient	23	11%
Normal shampoo	44	21.1%
‘Sensitive’ or fragrance free shampoo	101	48.3%
Water only	18	8.6%
Other	41	19.6%
Do you put bath emollients in your child's bath? (n=207)		
Yes, all the time	117	56.5
Yes, almost all the time	30	14.5
Yes, more than half the time	10	4.8
Less than half the time	14	6.8
Rarely	9	4.3
Never	27	13%
Do you think bath emollients help your child's eczema? (n=150)		
Yes	69	46%
No	12	8%
Unsure	69	46%

We carried out an online survey in order to inform study design, advertising through the National Eczema Society, Nottingham Support Group for Carers of Children with Eczema and Centre of Evidence-Based Dermatology. Carers were reporting about children with median age 4 and mostly moderate or severe eczema.

### Data collection methods

Data collection of participant-reported outcome measures will be carried out using online case report forms, or paper case report forms if participants prefer this. Participants will receive automated reminders by email and text/short message service (SMS). If they do not respond to this, then they will receive a telephone reminder at key time points, such as the 16-week questionnaire. Participants will also be sent a £10 gift voucher when the 16-week questionnaire is due and will be entered into a prize draw when they have completed the 52-week questionnaire.

Data collection from notes review will be carried out by practice staff using standardised questionnaires.

### Data management

Participants will enter their outcome data online into a validated database. Data will be stored on a secure server at the University of Southampton. Data collection from notes review and from participants who prefer to complete paper-based will be stored on paper in secure filing cabinets.

### Statistical methods

We will use repeated measures ANCOVA to explore whether there is a significant difference between mean POEM scores over the 16-week period in the intervention and standard care groups. The analysis will control for possible confounding effects of key covariates, such as baseline eczema severity and age of child. However, because ANCOVA relies on analysis of complete cases only, the levels of missing data will be reviewed and, if appropriate, the data will be analysed using mixed models instead, which allows incomplete cases to contribute to the analysis. There will be no interim analyses.

For the analysis of secondary outcomes, repeated measures analysis in line with that used for the primary outcome will be used for the monthly POEM measure up to 1 year. For other secondary outcomes, linear regression will be used for continuous outcomes if the assumptions are met. Otherwise non-parametric analyses will be used. Logistic regression will be used for dichotomous outcomes and a suitable count model, as determined by goodness of fit measures, for count data. All analyses will control for potential confounders.

Primary analyses will be carried out on an intention to treat basis, according to the CONSORT definition^31^ whereby all participants are included in the group to which they were assigned, whether or not they completed the intervention given to the group. We will consider carrying out a per protocol analysis in addition to this if monitoring reveals a substantial proportion are not following their treatment allocation (ie, intervention group stop using bath emollient or control group start bath emollient). We will carry out a sensitivity analysis based on prior belief in bath emollients, to explore whether this appears to have any influence on outcomes. The statistical methods will be finalised in a statistical analysis plan, which will be made available on the study website.

### Health economic analysis

The within trial economic analysis will include the primary economic evaluation which will be in the form of cost-effectiveness analysis. A secondary economic evaluation in the form of cost-utility analysis will be conducted using utility values obtained from the CHU-9D preference-based quality of life measure. Although the CHU-9D was not designed for use in very young children, it has been used among children aged less than 5 (M Chorozoglou, personal communication). All cost-effectiveness results will be presented on: (1) the cost-effectiveness plane, which captures the uncertainty around the results and shows the incremental cost and incremental effect of the comparison of interest in a two-dimentional plot, and (2) cost-effectiveness acceptability curves, which graphically represent the uncertainty, in terms of probabilities, regarding the cost-effectiveness of the new technology compared with the existing alternative. The cost-effectiveness unit of analysis will be: (1) the cost per unit change as measured by the primary outcome (POEM) at 16 weeks; (2) cost per exacerbation avoided over 1 year. If the latter is not possible then cost per unit score change of POEM measure at 1 year will be reported.

### Monitoring

The Trial Management Group is responsible for overseeing progress of the trial. An independent Trial Steering Committee will include Data Monitoring Committee roles because the trial is investigating bath emollients, used within their licensed range of indication, that are available without prescription and have been used for many years with no safety concerns. The Trial Steering Committee includes independent experienced triallist, medical statistician, dermatologist, and patient and public representative, following a standard National Institute for Health Research Health Technology Assessment (NIHR HTA) charter. No interim analyses are planned.

Information about expected adverse reactions to bath emollients which are listed in the Summary of Product Characteristics (ie, pruritus, reddening, itching, skin irritation, rash, accidental ingestion, slipping) will be collected in trial questionnaires (or withdrawal form, where applicable) and therefore need not be reported as an adverse event. Any unexpected adverse event which could reasonably have been caused by bath emollients will be reported.

## Discussion

This will be the first randomised controlled trial to investigate the role of bath emollients in the treatment of childhood eczema. The results of the trial will be important to families with eczema, primary care practitioners, dermatologists, dermatology nurses, guideline developers and decision-makers. We will disseminate the findings to all these groups as widely as possible.

This study has strengths and weaknesses. The impossibility of creating a convincing placebo for bath emollients means that it is not possible to carry out a blinded study. The central importance of having a participant-reported outcome measure for this pragmatic trial means that perceptions of outcome may be open to bias. We have mitigated for this by excluding participants who express a strong preference either for or against bath emollients, to the extent that they are unwilling to accept randomisation, and we ask participants randomised to the trial about prior belief in the effectiveness of bath emollients for eczema care, so that we can carry out a sensitivity analysis to explore the likely impact of such beliefs on the primary outcome.

A James Lind Alliance Priority Setting Partnership for eczema identified a key priority from patients and carers as ‘Which is the best way for people with eczema to wash?’^32^ The role of bath emollients in the treatment of childhood eczema was prioritised by the NIHR HTA Programme, and this study aims to find out whether bath emollients are beneficial for this condition.
